# Transcriptome Analysis of Fat Bodies from Two Brown Planthopper (*Nilaparvata lugens*) Populations with Different Virulence Levels in Rice

**DOI:** 10.1371/journal.pone.0088528

**Published:** 2014-02-12

**Authors:** Haixin Yu, Rui Ji, Wenfeng Ye, Hongdan Chen, Wenxiang Lai, Qiang Fu, Yonggen Lou

**Affiliations:** 1 State Key Laboratory of Rice Biology, Institute of Insect Sciences, Zhejiang University, Hangzhou, China; 2 Research and Development Center of Rice Production Technology, China National Rice Research Institute, Hangzhou, China; The Scripps Research Institute, United States of America

## Abstract

**Background:**

The brown planthopper (BPH), *Nilaparvata lugens* (Stål), one of the most serious rice insect pests in Asia, can quickly overcome rice resistance by evolving new virulent populations. The insect fat body plays essential roles in the life cycles of insects and in plant-insect interactions. However, whether differences in fat body transcriptomes exist between insect populations with different virulence levels and whether the transcriptomic differences are related to insect virulence remain largely unknown.

**Methodology/Principal Findings:**

In this study, we performed transcriptome-wide analyses on the fat bodies of two BPH populations with different virulence levels in rice. The populations were derived from rice variety TN1 (TN1 population) and Mudgo (M population). In total, 33,776 and 32,332 unigenes from the fat bodies of TN1 and M populations, respectively, were generated using Illumina technology. Gene ontology annotations and Kyoto Encyclopedia of Genes and Genomes (KEGG) orthology classifications indicated that genes related to metabolism and immunity were significantly active in the fat bodies. In addition, a total of 339 unigenes showed homology to genes of yeast-like symbionts (YLSs) from 12 genera and endosymbiotic bacteria *Wolbachia*. A comparative analysis of the two transcriptomes generated 7,860 differentially expressed genes. GO annotations and enrichment analysis of KEGG pathways indicated these differentially expressed transcripts might be involved in metabolism and immunity. Finally, 105 differentially expressed genes from YLSs and *Wolbachia* were identified, genes which might be associated with the formation of different virulent populations.

**Conclusions/Significance:**

This study was the first to compare the fat-body transcriptomes of two BPH populations having different virulence traits and to find genes that may be related to this difference. Our findings provide a molecular resource for future investigations of fat bodies and will be useful in examining the interactions between the fat body and virulence variation in the BPH.

## Introduction

The insect fat body, which consists mainly of adipocytes, is a central storage depot for excess nutrients. It not only controls the synthesis and use of energy reserves, lipid and glycogen, it also participates in multiple biochemical functions of intermediate metabolism, such as lipid and carbohydrate metabolism, amino acid and nitrogen metabolism, and protein synthesis [1]. Moreover, the insect fat body is involved in immunity, the production of antimicrobial peptides, and the detoxification of nitrogen metabolites [2]. That the insect fat body is an organ of great biosynthetic and metabolic activity has also been confirmed by research on the fat body transcriptomes of several insect species, such as *Drosophila melanogaster* Meigen (Diptera: Drosophilidae), *Glossina morsitans morsitans* Westwood (Diptera: Glossinidae), *Bombyx mori* L. (Lepidoptera: Bombycidae), and *Aedes aegypti* (L.) (Diptera: Culicidae) [3]–[6]. In addition, the fat bodies in certain insect species, such as cockroaches, beetles, and some Hemiptera (e.g. planthoppers, aphids), contain mycetocytes [7]. These mycetocytes have symbiotic microorganisms which are supposed to produce essential components that insects cannot produce alone and which may play a role in the formation of biotypes or virulent populations of host insects, such as whiteflies, aphids and the brown planthopper (BPH), *Nilaparvata lugens* (Stål) [7]–[11]. Therefore, the insect's fat body plays essential roles in its life cycle and in its interactions with plants. However, whether differences in fat body transcriptomes exist between insect populations with different virulence levels and whether these differences are related to insect virulence remain unanswered questions.

The BPH, one of the most serious pests of rice in Asia, sucks sap from rice phloem; this stunts plant growth and transmits plant viruses, such as the rice ragged stunt virus and the rice grassy stunt virus [12]–[13]. At present, chemical insecticides are still the first choice for BPH management. However, the long-term application of insecticides has caused the BPH to develop resistance, which may result in its resurgence and in environmental pollution [13]. Another strategy for BPH control is to cultivate resistant rice varieties. However, the BPH has shown repeatedly that it can ‘adapt’ to these resistant rice varieties by establishing different virulent populations [14]. There are thought to be some differences in morphological features, DNA polymorphisms, and the composition of microbial symbionts among virulent BPH populations [11], [15]–[17]. However, the mechanisms underlying changes in BPH virulence are not clear. Investigating the molecular response of the BPH to three resistant rice varieties, Yang *et al.* (2006) identified 61 differentially expressed genes in BPH that were involved in signaling, stress response, gene expression regulation, detoxification and metabolism [18]. The ability to synthesize or acquire rare amino acids was found to be important for the ability of the BPH to adapt to resistant rice varieties and to form new virulent populations [14]. In the fat body of the BPH, there are a large number of microbial symbionts. That the species and/or the numbers of yeast-like symbionts (YLSs) and bacterial symbionts differed substantially among the BPH populations with different levels of virulence suggests these symbionts may play a role in the evolution of BPH virulence and in the synthesis and use of energy reserves, intermediate metabolism, immune response and plant-insect interactions [7], [11], [16], [19]. Although the transcriptomes or expressed sequence tags (ESTs) of some BPH tissues, such as midgut, salivary gland, head, abdomen, thorax, testis, and egg, have been reported, the fat body transcriptome has not been investigated [13], [20]. Moreover, whether there is a difference in fat body transcriptomes between BPH populations with different virulence levels remains unclear.

To explore these issues, we compared the fat body transcriptomes of two BPH populations, the avirulent TN1 population and the virulent Mudgo (M) population. A total of 33,776 and 32,332 unigenes were identified in the TN1 and M populations, respectively, and 7,860 differentially expressed genes were identified between them. Moreover, enrichment analysis of Kyoto Encyclopedia of Genes and Genomes (KEGG) pathway for differentially expressed genes showed that 17 pathways related to metabolism and immunity were significantly enriched. In addition, 339 genes from YLSs and the endosymbiotic bacteria *Wolbachia* were also identified. Among these genes, 105 showed altered expression. These results may help elucidate the role of the fat body in the life cycle of the BPH, BPH-rice interactions, and in BPH virulence variation.

## Materials and Methods

### BPH cultures, fat body collection, and RNA isolation

Two rice varieties, a susceptible TN1 variety and Mudgo, which carries the resistance gene *Bph1*, were used to maintain two BPH populations, designated TN1 (avirulent population) and M (virulent population), respectively, at the Institute of Insect Sciences, Zhejiang University. The insectary was set at 27±1°C and 70±10% relative humidity under a 14:10 h light/dark photoperiod. The original insects were provided by the Chinese National Rice Research Institute (Hangzhou, China). From each population, TN1 and M, 150 adult females were collected and placed in a Petri dish on ice. Their fat bodies were dissected using microforceps and immediately transferred to a diethylpyrocarbonate (DEPC)-treated phosphate buffer saline solution (pH 7.2). Total RNA was isolated from the fat bodies using the SV Total RNA Isolation System Kit (Promega, Fitchburg, WI, USA) according to the manufacturer's protocol. The concentration and quality of total RNA were determined by a NanoDrop spectrophotometer (Thermo Fisher, Waltham, MA, USA).

### cDNA library preparation and Illumina sequencing

The fat body cDNA library was prepared using a SMARTer™ PCR cDNA Synthesis Kit (Clontech, Mountain View, CA, USA) and an Advantage 2 PCR Kit (Clontech). After the end-repair and ligation of adaptors, cDNA products were amplified by PCR and purified using the QIAquick PCR Purification Kit (Qiagen, Hilden, Germany) to create cDNA libraries. The cDNA libraries were sequenced on the Illumina sequencing platform at BGI-Shenzhen (Shenzhen, China). The raw reads were generated using Solexa GA pipeline 1.6 (Illumina). After the removal of adaptor sequences, empty reads, and low quality reads, the processed reads were assembled using SOAP *de novo* software and clustered with TGI Clustering tools [21]–[22]. All raw transcriptome data were deposited in the SRA database (NCBI) with accession number SRX360412 (TN1 population) and SRX360414 (M population). The generated unigene sequences were analyzed by searching the GenBank and SwissProt databases with the BLASTX algorithm (http://www.ncbi.nlm.nih.gov/) [23]. GO and KEGG Orthology annotations of the unigenes were determined using the Blast2go (http://www.blast2go.org/) and Inter-ProScan software (http://www.ebi.ac.uk/Tools/pfa/iprscan/).

### Analysis of differential gene expression

Fat body genes that were differentially expressed between TN1 and M populations were identified using a table of counts constructed with fragments per kb per million fragments (FPKM) values, which adjusted the number of fragments by the total number of fragments mapped and the length of the gene [24], [25]. The false discovery rate (FDR) was used to determine threshold *P*-values in the multiple test and analysis. An FDR<0.001 and an absolute value of the log_2_ ratio>1 provided significance thresholds for gene expression differences.

To confirm the results of the FPKM analysis, the expression levels of 28 randomly selected fat body genes were measured in TN1 and M populations by QRT-PCR. Total RNA from each sample (fat bodies of about 120 females per sample) was extracted using the SV Total RNA Isolation System kit (Promega). The concentration of each RNA sample was adjusted to 1 µg/µl with nuclease-free water and total RNA was reverse-transcribed in a 20 µl reaction using the Primescript™ RT reagent kit (TaKaRa). QRT-PCR was performed in the CFX96™ Real-Time System (Bio-Rad, Hercules, CA, USA) with SYBR green detection under the following conditions: 95°C for 3 min, and 40 cycles of 95°C for 5 s, 60°C for 15 s, following by melting curve generation at 65 to 95°C. Primers used for QRT-PCR are shown in Table S1. Each gene was analyzed in three biological replications, after which the average threshold cycle (C_t_) per sample was calculated. As an endogenous control, the expression of 18S rRNA was measured in parallel, and a no-template-control sample (nuclease-free water) was also included to detect contamination and determine the degree of dimer formation in the experiment. Finally, the relative expression levels were calculated using the 2^−ΔΔCt^ method.

### Identification of statistically enriched KEGG pathways

The differentially expressed genes were used for KEGG pathway enrichment analysis using the hypergeometric test to measure significantly enriched terms. The formula was:
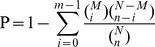



In this equation, *N* indicates the number of genes with KO annotations and *n* the number of differentially expressed genes in *N*. The variables *M* and *m* represent the numbers of genes and differentially expressed genes, respectively, in each KO term. The threshold to determine significant enrichment of the gene sets was corrected to *P*-value ≤0.05.

## Results and Discussion

### Illumina sequencing and read assembly

The fat body cDNA libraries of the TN1 and M populations were sequenced using the Illumina platform, resulting in 37,475,676 and 35,867,838 reads, respectively, with a total of 6,600,916,260 nucleotides. After cleaning and quality checks, short sequences were assembled, resulting in 89,748 TN1 and 91,262 M population contigs. Using paired-end joining and gap-filling, the TN1 and M contigs were assembled into 66,349 and 66,367 scaffolds, respectively, which were then clustered into 33,776 and 32,332 unigenes, respectively (Table 1). After clustering the scaffolds together with nucleotide sequences available at NCBI, sequence data from the two libraries were combined, and 42,621 unigenes were finally obtained; collectively, the genes had a mean size of 517 bp (Table 1). The length distribution of total unigenes had similar patterns between TN1 and M samples, suggesting there was no bias in the construction of the cDNA libraries (Figure S1).

**Table 1:** Summary statistics for the fat body transcriptomes of two brown planthopper populations.

	TN1 population	M population	Combined
Total number of reads	37,475,676	35,867,838	73,343,514
Total base pairs(bp)	3,372,810,840	3,228,105,420	6,600,916,260
Average read length(bp)	90	90	90
Average read length(bp)	90	90	90
Total number of contigs	89,748	91,262	181,010
Mean length of contigs	282	274	278
Total number of scaffolds	66,349	66,367	132,716
Mean length of scaffolds	394	392	393
Total unique sequences	33,776	32,332	42,621
Mean length of unigene(bp)	656	676	517

### Annotation of fat body transcripts

**Figure 1:**
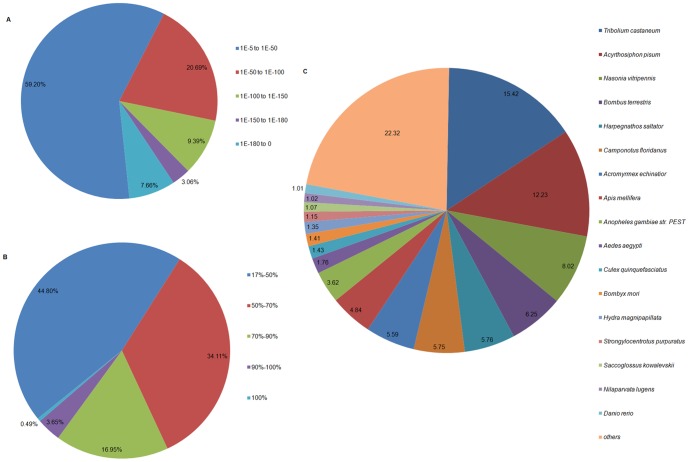
Characteristics of the homology search of Illumina sequences against the Nr database. (A) E-value distribution of BLAST hits for each unique sequence with a cut-off E-value of 1.0E^−5^. (B) Similarity distribution of the top BLAST hits each sequence. (C) Species distribution is shown as a percentage of the total homologous sequences with an E-value of at least 1.0E^−5^. We used the first hit for each sequence in the analysis.

For functional annotation, we searched all of the reference sequences from the cDNA libraries of the TN1 and M populations using BLASTX against the non-redundant (nr) NCBI protein database with a cut-off E-value of 10^−5^. A total of 19,696 (46.21% of all distinct sequences) unigenes provided a BLAST result (Table S2). The E-value distribution of the best hits against the nr database showed that 21.11% of the sequences have strong homology (E-value<1.0E^−100^), whereas 79.89% of the homolog sequences ranged between 1.0E^−5^ to 1.0E^−100^. The E-values of most of the sequences ranged from to 1.0E^−5^ to 1.0E^−50^ (Figure 1A). The similarity distribution indicated that 21.09% of the unique sequences with best hits had a similarity higher than 70%, while 78.91% of the hits had a similarity ranging from 17% to 70% (Figure 1B). The species distribution of the best match results for each sequence is shown in Figure 1C and Table S3. The unigenes of the fat bodies showed 15.42% homology with the genes of the red flour beetle, *Tribolium castaneum* (Herbst), and 12.23%, 8.02% and 6.25% homology with the genes of the *Acyrthosiphon pisum* (Harris), *Nasonia vitripennis* (Walk.) and *Bombus terrestris* (L.), respectively. A similar result was also reported by Bao *et al.* [13]. Further research should explore why the highest percentage of unique sequences matched the genes of *T. castaneum*, a coleoptera beetle, rather than the genes of *A. pisum*, a hemiptera aphid more closely related to the BPH.

### GO and KEGG orthology classifications

**Figure 2:**
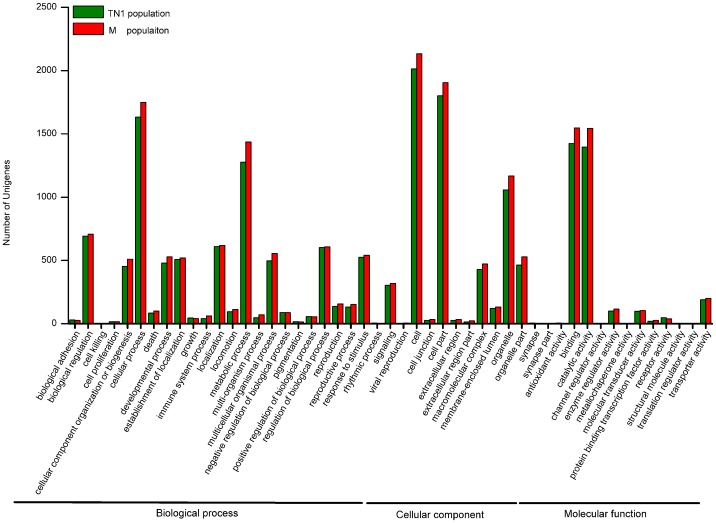
Gene ontology (GO) classifications of fat-body genes of TN1 and Mudgo (M) populations. The *x*-axis shows subgroups of molecular functions from GO classifications and the *y*-axis shows the number of the matching unigenes in a category.

Gene Ontology (GO) assignments were used to classify the functions of predicted BPH fat body unigenes. Among the 33,776 and 32,332 annotated unigenes in the fat bodies of TN1 and M populations, respectively, 19,027 (56.33%) and 17,635 (54.54%) of the unigenes could be annotated in GO assignments based on sequence homology, respectively (Table S4). When compared with the TN1 population, the M population had a very similar GO distribution. For both libraries, the ‘biological processes’ category most represented was ‘cellular process’, and the ‘cellular component’ category most represented was ‘cell’, whereas ‘binding and catalytic activity’ was the most represented ‘molecular function’ category (Figure 2).

**Figure 3:**
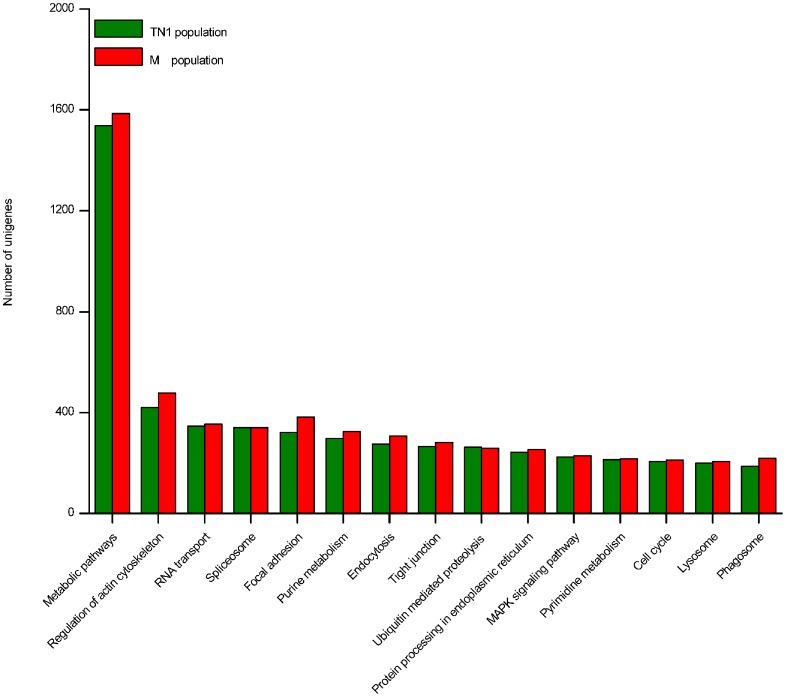
KEGG pathway distributions of unigenes from fat bodies of TN1 and Mudgo (M) populations. The top fifteen pathways (excluding disease related) with highest percentages of unigenes mapped to are shown.

Kyoto Encyclopedia of Genes and Genomes (KEGG) is a database of biological systems that integrates genomic, chemical and systemic functional information. To investigate which biological pathways were active in the fat bodies, all of the sequences were assigned to the reference canonical pathways in the KEGG. A similar distribution of biological pathways for TN1 and M populations was also found by KEGG mapping, and a total of 20,920 unigenes from TN1 population and 22,427 unigenes from M population were mapped separately to 240 and 242 pathways in total (Figure 3). The fat body was a dynamic tissue with the functions of multiple metabolic, including lipid and carbohydrate metabolism, amino acid and nitrogen metabolism, and protein synthesis [1]. Consequently, a high level of metabolic activity took place in this organ. Obviously, among these pathways, ‘metabolic pathways’ was the most dominant in the fat bodies (1,538 unigenes from TN1 population and 1,586 unigenes from M population, Figure 3, in pathways associated with human diseases were excluded). As the center of the multiple metabolisms, the fat bodies should be active in protein synthesis and catabolism, lipid metabolism, carbohydrate metabolism, xenobiotic and energy metabolism. In addition, in the ‘protein processing in endoplasmic reticulum’ pathway contained many sequences (243 unigenes from TN1 population and 254 unigenes from M population), which was related to the formation and transport of proteins and amino acids. Moreover, the ‘lysosome’ pathway (199 unigenes in TN1 population and 206 unigenes in M population) and the ‘phagosome’ pathway (188 unigenes in TN1 population and 219 unigenes in M population), two major pathways played important roles in innate immune responses in BPH. The results of GO annotations and KEGG mapping indicated that the fat bodies might be active in metabolism and immune.

### Transcripts related to symbiotic microorganisms

**Figure 4:**
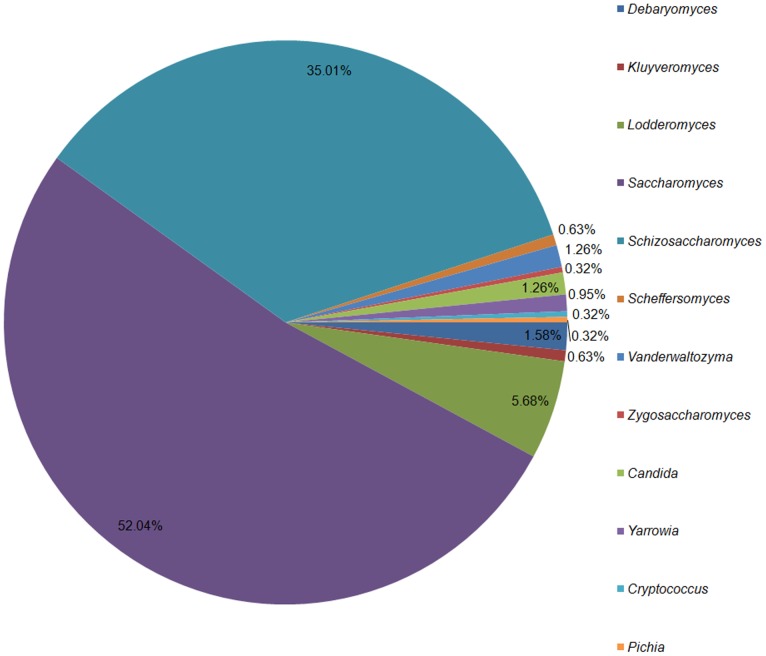
The genera of yeast-like symbionts in the fat body of the brown planthopper *Nilaparvata lugens*.

Symbiotic microorganisms of phloem-feeding insects may improve diet quality by synthesizing essential amino acids lacking in their host insect's diet [26]. In our fat body transcriptomes, we found many transcripts related to YLSs and *Wolbachia*. YLSs are microbial symbionts that reside intracellularly in the BPH's fat body cells and provide some nutrients for their hosts, such as sterols, vitamins, and rare amino acids [11], [23]. In the previous study, the YLSs of the BPH mainly consisted of fungal species from the Ascomycetes, Pyrenomycetes, Blastomycetes, and Agaricostibomycetes, including *Pichia*-like symbionts, *Cryptococcus*-like symbionts, *Yarrowia*-like symbionts, *Sterigmatomyces*-like symbionts, *Hypomyces*-like symbionts, and *Candida*-like symbionts [27]–[31]. In this study, we retrieved 317 unigenes that showed homology to genes of fungal species from 12 genera (Blastx search, E-value <10^−5^) (Figure 4). Four of these genera, *Pichia*, *Cryptococcus*, *Yarrowia* and *Candida*, had been previously reported in the BPH, whereas this is the first identification and report of the other eight genera, *Debaryomyces*, *Kluyveromyces*, *Lodderomyces*, *Saccharomyces*, *Schizosaccharomyces*, *Scheffersomyces*, *Vanderwaltozyma*, and *Zygosaccharomyces*, in the BPH. Although the data are too preliminary to state definitively that all these YLSs are present in the BPH, this information will be useful for further research on BPH endosymbionts.

*Wolbachia* is a group of obligatory, intracellular, Gram-negative bacteria that infect a wide range of arthropods and nematodes [32]. *Wolbachia* can be transferred horizontally between different insect species and affect the host's sexual reproduction, cytoplasmic incompatibility, and immune responses [32]–[33]. In BPH *Wolbachia* are reported to be widely distributed in the various populations [16]. Here, we annotated 22 *Wolbachia* unigenes, which were mainly involved in cell processes, the biosynthesis of cofactors and protein metabolism, and transport (Table S5).

### Global patterns of differentially expressed genes

**Figure 5:**
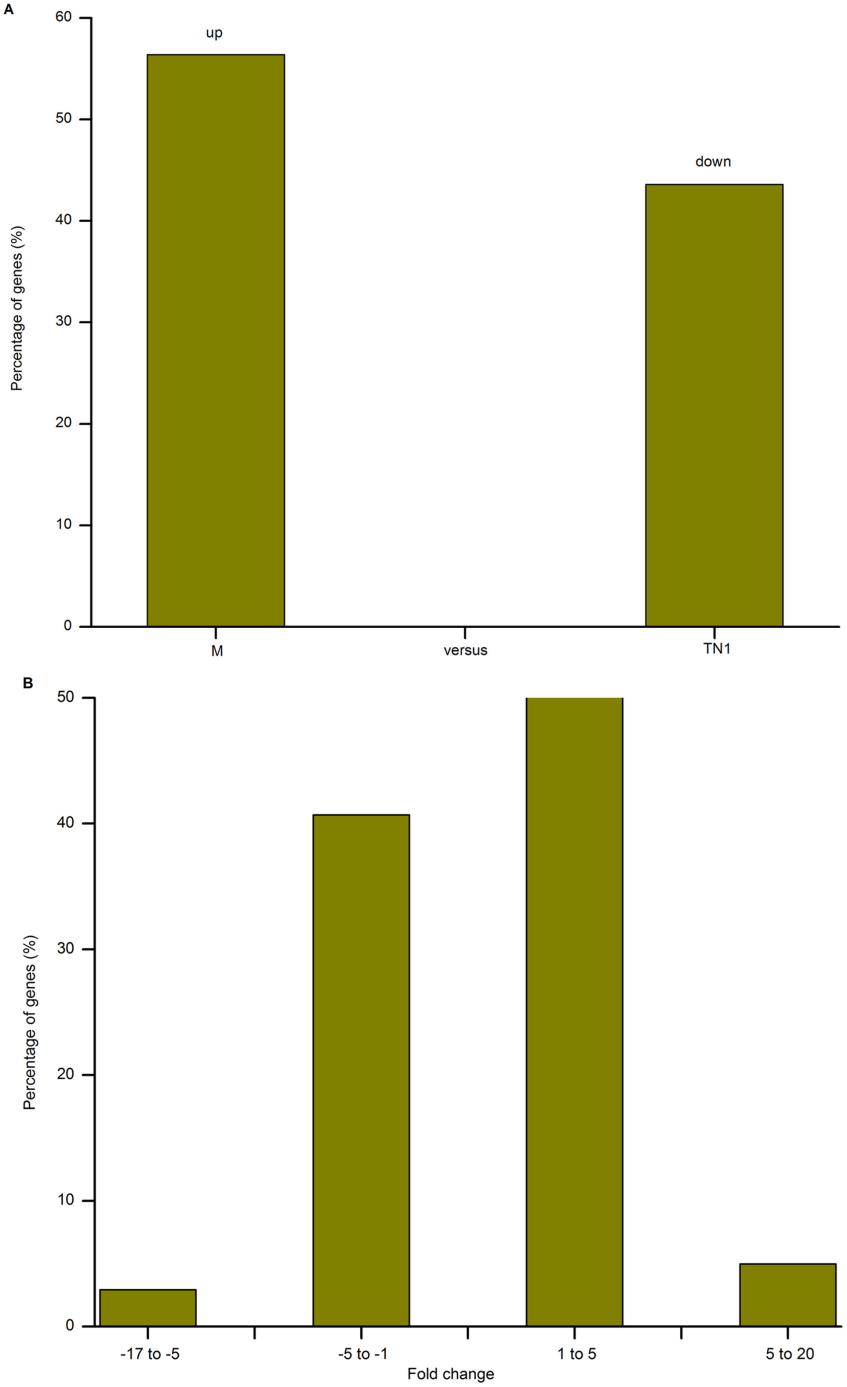
Summary of differentially expressed genes from fat bodies of TN1 and Mudgo (M) populations. (A) Summary of the percentage of differentially expressed genes in the fat bodies of TN1 and M populations. (B) Fold change distribution of differentially expressed genes.

In total, 7,860 significantly differentially expressed genes were found between the fat body transcriptomes of the two populations. Among these genes, 56.40% (4,433 genes) had higher and 43.60% (3,427 genes) had lower levels of transcripts in the M population than in TN1 population (Figure 5A). The detected fold changes (log_2_ ratio) of gene expression ranged from −17 to +20, and more than 80% of the genes were up- or down-regulated between 1.0- and 5.0-fold (Figure 5B). Moreover, among the differentially expressed genes, 3,858 (49.1%) were annotated, including 2,765 up-regulated genes in the M population. To validate these gene expression data, we compared the expression profiles of the fat bodies of the populations using quantitative real-time PCR (QRT-PCR). Of the 28 randomly selected genes, all showed similar fold differences between the types of analyses, indicating that our results were reliable (Table S6).

**Figure 6:**
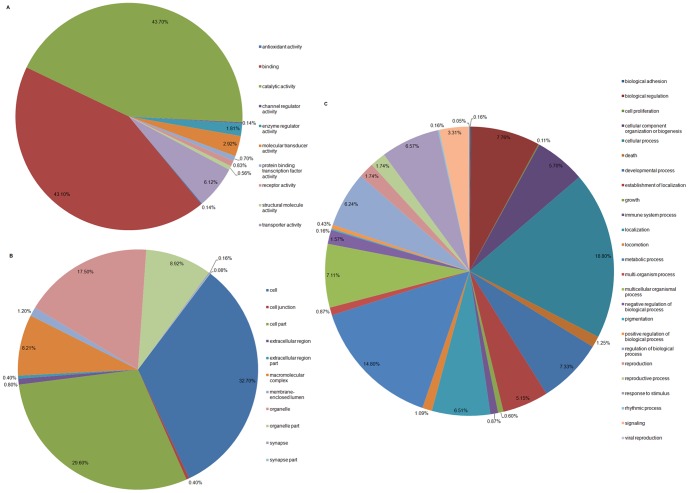
Distribution of significantly differentially expressed transcripts in gene ontology (GO) subclasses. (A) Biological process. (B) Molecular function. (C) Cellular component.

The differentially expressed genes were organized according to 46 functional groups, such as ‘biological process’ (1,843 genes), ‘molecular function’ (719), and ‘cell component’ (1,255). The ontology distributions are shown in Figure 6. In the ‘biological process’ category, the top three classifications were ‘cellular process’ (346 genes, 18.77%), ‘metabolic process’ (272, 14.76%), and ‘biological regulation’ (143, 7.76%) (Figure 6A, Table S7). In ‘molecular function’, most genes were related to ‘catalytic activity’ (314, 43.67%), ‘binding’ (310, 43.12%), and ‘transporter activity’ (44, 6.12%) (Figure 6B, Table S7). The ‘cell’ (411, 32.75%) classification was the main group in ‘cell component’ (Figure 6C, Table S7). The differentially expressed genes may affect the physiological and biochemical processes in the fat bodies of these populations; however, further functional studies must be performed to validate this hypothesis.

To gain insight into the dominant biological pathways of the differentially expressed genes that mapped to KEGG orthologs, a hypergeometric test was performed to explore statistically enriched pathways. Consequently, 27 enriched pathways (*P*≤0.05) were identified (Table 2; pathways associated with human disease were excluded). Twelve gene sets were correlated with ‘metabolism’, five with ‘immunity’, and 10 with other pathways (Table 2). The results indicated that the differentially expressed genes might be mainly active in metabolism and immunity.

**Table 2:** Enrichment analysis of KEGG pathways in the fat body transcriptomes of two brown planthopper populations.

KEGG Pathway	*P*-value	Total^1^	Up-regulated^2^	Down-regulated^3^
**Metabolism**				
**Carbohydrate metabolism**				
Glycolysis/Gluconeogenesis	5.71E-07	44	37	7
Citrate cycle (TCA cycle)	3.40E-03	30	28	2
Pentose phosphate pathway	7.56E-03	20	16	4
Amino sugar and nucleotide sugar metabolism	4.95E-03	34	31	3
Pyruvate metabolism	1.02E-03	26	24	2
**Lipid metabolism**				
Steroid biosynthesis	2.46E-02	5	5	0
Glycerolipid metabolism	3.59E-04	27	21	6
Alpha-Linolenic acid metabolism	3.69E-02	11	8	3
**Amino acid metabolism**				
Tyrosine metabolism	6.38E-04	33	29	4
Arginine and proline metabolism	1.16 E-02	30	25	5
Valine, leucine and isoleucine biosynthesis	1.2 E-02	13	12	1
**Xenobiotics biodegradation and metabolism**				
Drug metabolism - other enzymes	4.58 E-02	22	17	5
**Immune**				
**Cellular and humoral immune responses**				
Lysosome	4.85 E-02	48	38	10
Phagosome	3.20E-08	88	75	13
Complement and coagulation cascades	4.26 E-03	20	19	1
**Immune systems and signaling transduction**				
Toll-like receptor signaling pathway	4.60 E-02	14	11	3
JAK-STAT signaling pathway	4.28 E-02	24	18	6
**Others**				
Regulation of actin cytoskeleton	9.76E-06	164	132	32
Focal adhesion	3.67 E-03	127	99	28
Tight junction	3.81 E-04	98	76	22
ECM-receptor interaction	2.44 E-03	58	49	9
Cytokine-cytokine receptor interaction	7.50 E-03	23	20	3
Olfactory transduction	7.40 E-03	38	34	4
Fat digestion and absorption	3.33 E-04	32	19	13
Endocrine and other factor-regulated calcium reabsorption	3.12 E-02	28	23	5
Vitamin digestion and absorption	1.12 E-02	27	11	16
Calcium signaling pathway	4.08 E-02	69	56	13

1 Number of differentially expressed genes in fat bodies belonging to each KEGG pathway.

2 Number of genes up-regulated in the M population relative to the TN1 population in each KEGG pathway.

3 Number of genes down-regulated in the M population relative to the TN1 population in each KEGG pathway.

### Differentially expressed genes related to metabolism

The insect fat body is an organ of great biosynthetic and metabolic activity [1]. The KEGG pathway enrichment analysis of differentially expressed genes showed that 12 pathways related to metabolism were enriched. These were involved in carbohydrate metabolism, lipid metabolism, amino acid metabolism, and the biodegradation and metabolism of xenobiotics (Table S8).

### Carbohydrate metabolism

For carbohydrate metabolism, most of the genes in the five significantly enriched pathways, glycolysis/gluconeogenesis (37 genes, 84.09%), citrate cycle (TCA cycle) (28, 93.33%), pentose phosphate (16, 80.0%), amino sugar and nucleotide sugar metabolism (31, 91.18%), and pyruvate metabolism (24, 92.31%), had higher transcript levels in the M population than in the TN1 population (Table S8). Carbohydrates, especially sucrose, are the main chemical components in the phloem sap of rice and are essential for the phloem-sucking insects as phagostimulants as well as nutrients [33]–[34]. Carbohydrates are a major energy source for the BPH, and their carbon skeletons can contribute to amino acid production [35]–[36]. In addition, the number of total carbohydrates, especially the soluble sugar content, in highly resistant rice varieties is lower than the number in susceptible varieties [37]. Thus, a high level of carbohydrate metabolism in the M population might not only compensate for insufficient nutrients in the phloem sap of resistant rice varieties but also be helpful for the adaptation of BPH to resistant rice varieties.

### Lipid metabolism

In insects, lipids stored as droplets – a triglyceride core surrounded by a layer of phospholipids and embedded proteins – represent the major component of the fat body and the main source of metabolic fuel [1]. Lipid metabolism is an active process in the fat body of the BPH. In our enrichment analysis of KEGG pathway, most of the genes in the three enriched pathways involved in lipid metabolism, including steroid biosynthesis (5 genes, 100.0%), glycerolipid metabolism (21, 77.78%), and alpha-linolenic acid metabolism (8, 72.73%), had higher transcript levels in the M population than in the TN1 population (Table S8). Seven unique genes in these pathways encoded the same enzyme, triacylglycerol lipase, which was a hormonally regulated enzyme that catalyzed the hydrolytic release of fatty acid from carbon 1 or 3 of the glycerol moiety [38]. Four unique genes encoded phospholipase A2 (PLA_2_), which was able to catalyze the first step in eicosanoid biosynthesis by hydrolyzing arachidonic acid (AA) from cellular phospholipids (PLs). Eicosanoids were crucial mediators of visible cellular immune mechanisms, such as phagocytosis and nodulation [39]. With the exception of these two enzymes, the enzymes encoded by differentially expressed genes in the three enrichment pathways might contribute to the change in virulence between the two populations.

### Amino acid metabolism

Phloem sap is rich in simple sugars but low in nitrogenous organic compounds, especially amino acids. For example, in rice phloem sap, sucrose and amino acids make up about 17 to 25% and 3 to 8% (w/v), respectively [40]. Nitrogen availability is important to phloem-feeding insects, and free amino acids are the most important nitrogenous compounds available in phloem sap [41]. However, the amount of free amino acids in the resistant rice variety was significantly lower than that in susceptible varieties [37]. Thus, the M population of BPH might modulate the levels of amino acid metabolism to adapt to the resistant rice variety. Indeed, tyrosine metabolism, arginine and proline metabolism, and valine, leucine and isoleucine biosynthesis were the three most dominant amino acid metabolic pathways, and most of the unigenes had higher transcript levels in the M population than in the TN1 population (Table S8). Among them, 33 genes were involved in an enriched pathway, tyrosine metabolism, which plays a central role in the sclerotization or tanning of the insect's cuticle [42]. Thirty genes were involved in arginine and proline metabolism, including genes coding for arginase, ornithine δ-transaminase, Δ1-pyrroline-5-carboxylate reductase, Δ1-pyrroline-5-carboxylate synthase, and glutamate-5-semialdehyde dehydrogenase. In general, arginine, proline and glutamate are derived from α-ketoglutarate. Arginine can be converted to proline when catalyzed by arginase, ornithine δ-transaminase and Δ1-pyrroline-5-carboxylate reductase [41]. Meanwhile, Δ1-pyrroline-5-carboxylate reductase, Δ1-pyrroline-5-carboxylate synthase, and glutamate-5-semialdehyde dehydrogenase are thought to be components of the catabolic pathway for the conversion of glutamate into proline. Proline is a major substrate used in insect flight metabolism [41]. Since glutamate is one of the dominant amino acids in rice phloem sap [40], the increase in putative enzymes involved in the conversion of glutamate into proline suggested that members of the M population might improve their absorption and use for amino acids. In addition, valine, leucine, and isoleucine are three branched-chain amino acids [41]. In this study, 14 genes were involved in the pathway that enriched the biosynthesis of valine, leucine, and isoleucine. Among them, one up-regulated gene in the M population encoded the branched-chain amino acid transaminase, which catalyzed the final reaction in the production of each of the three branched-chain amino acids.

### Xenobiotics biodegradation and metabolism

In insects, cytochrome P450 monooxygenases (P450s), glutathione S-transferases (GSTs), and carboxylesterases (COEs) are members of the three major multigene enzyme families responsible for xenobiotic metabolism [43]. Here we found one enrichment pathway, drug metabolism, contained other enzymes involved in the biodegradation and metabolism of xenobiotics (Table S8). In this pathway, 17 genes (77.27%) had higher and 5 (22.73%) had lower transcript levels in the M population than in the TN1 population. Among them, only one gene (a higher transcript level in M population) encoded a cytochrome P450, which was grouped in the CYP3 clade. The genes from the CYP3 clade might participate in xenobiotic metabolism, and they evolved very rapidly [44]. In insects, COEs can be divided into 13 clades, of which clades E and F were represented in the pathway of xenobiotic metabolism [43]. Compared to those in the TN1 population, eight COE genes in the M population had higher and one had lower transcript levels. These genes include four juvenile hormone esterase sequences (clade F) and five carboxylesterase sequences (clade E) (Table S8). Meanwhile, four genes encoding GSTs were also determined: three had higher and one had lower levels of transcripts in the M population than in the TN1 population. The abundant detoxification-related differentially expressed genes suggested that the levels of xenobiotic biodegradation and metabolism changed between the two populations, which might be related to the virulence variation of the BPH.

The above analysis indicated that the difference in ‘metabolism’ might contribute to the virulence variation of the two populations. However, more evidence is required to prove this hypothesis.

### Differentially expressed genes related to immunity

Insects lack adaptive immunity, relying instead on both constitutive and inducible defense mechanisms to combat diverse microbial infections [4]. The results of a KEGG pathway enrichment analysis showed the five pathways related to immunity were enriched. These pathways are involved in lysosome, phagosome, complement and coagulation cascades, the JAK-STAT signaling pathway, and the Toll-like receptor signaling pathway (Table S9).

### Cellular and humoral immune responses

For insects, cellular and humoral responses are the major effector response systems against microbial infection [45]. In our pathway analysis, lysosomes and phagosomes were assigned to the cellular response, while the complement and coagulation cascades were assigned to the humoral response. Interestingly, among the differentially expressed genes, many genes related to cellular and humoral immune responses had higher transcript levels in the M population than in the TN1 population. Lysosomes and phagosomes have been shown to play a direct antiviral role [46]. In the M population, 38 genes (79.17%) involved in lysosome functions and 75 genes (85.23%) involved in phagosome functions had higher levels of transcripts compared to those in the TN1 population, strongly suggesting that these pathways were activated in the virulent M population. In addition to the up-regulation of most of the genes involved in the cellular response, significant numbers of genes involved in the humoral response, such as complement and coagulation cascades, were also up-regulated in the M population (Table S9). This suggests that some substance or microorganism in the Mudgo rice varieties activates both the cellular and humoral immune response of the BPH, which might result in increases in the levels of immunity in the M population with high virulence.

### Immune systems and signaling transduction

In our pathway enrichment analysis, two pathways related to the immune systems and signal transduction, the Toll-like receptor and JAK-STAT signaling pathways, were enriched (Table S9).

Components of Gram-positive bacteria and fungi can activate the Toll-like receptor pathway. It is triggered by the binding of a cytokine named Spätzle, which is necessary and sufficient for the production of antimicrobial peptides in the fat body of insects [47]. In our Toll-like receptor pathway, 11 genes (78.57%) in the M population were up-regulated and three (21.43%) down-regulated, including two Toll-like receptor genes and one Pellino gene (Table S9). The function of the Toll-like receptor might be to relay intracellular signals by binding Spätzle in the fat body [47]. Pellino proteins are intracellular signaling molecules in the Toll-like receptor pathway. As scaffolding proteins, the function of the Pellino gene is to facilitate the release of phosphorylated IRAK from the receptor [48].

The JAK/STAT pathway consists of four major components: the ligand UPD, the receptor Domeless, the Janus kinase (JAK), and the signal transduction and activators of transcription (STAT). In *Drosophila*, the JAK-STAT pathway is usually involved in the differentiation of hemocytes and resistance to bacterial or fungal infection [49]. In our JAK-STAT signaling pathway, compared with those in TN1 population, 18 genes (75.0%) in M population had higher and six genes (25.0%) had lower transcript levels, including three genes encoding suppressor of cytokine signaling (SOCS). It has been reported that SOCS proteins, which are induced by cytokine signaling, constitute a class of negative regulators for the JAK-STAT pathway [50].

The number of differentially expressed genes in the enriched pathways involved in immunity suggests the mechanism of BPH virulence is more complicated than previously believed and will require further investigation.

### Differentially expressed genes related to symbiotic microorganisms

The 85 genes that showed homology to genes of *Debaryomyces*, *Pichia*, *Kluyveromyces*, *Lodderomyces*, *Saccharomyces*, and *Schizosaccharomyces* were differentially expressed between the two BPH populations, and 57 genes (67.06%) had higher mRNA levels in the M population than in the TN1 population (Table S5). Among these genes, five were involved in protein transport, including an intracellular protein transport protein, a vacuolar amino acid transporter, and a Golgin IMH1. In addition, one gene was involved in ribosome biogenesis. These results suggest that the M population might have more protein transport activity than the TN1 population, which could be linked with the virulence variation of the BPH.

We annotated 22 *Wolbachia* genes, and 20 genes (90.9%) had higher levels of mRNA in the M population than in the TN1 population (Table S5). The functional annotation of *Wolbachia* genes could be classified into three major functional categories: (1) Genes essential for cell processes such as ribosomal assembly and cell division, including a 30S ribosomal protein, 50S ribosomal protein and the cell division protein FtsZ; (2) Genes responsible for *de novo* biosynthesis of cofactors and protein metabolism, including ATP-specific succinyl-CoA synthetase, elongation factor Tu 2, translation initiation factor IF-2, and cytosol aminopeptidase; and (3) Genes involved in transport, including a set of chaperones (DnaK, GroES, and a cold shock protein). The gene encoding the cell division protein FtsZ had a higher mRNA level in the M population compared to the TN1 population, which inferred that *Wolbachia* in the M population might have higher levels of cell division activity than those in the TN1 population. The above-identified *Wolbachia* genes might reveal the molecular mechanism of how an endosymbiotic bacterium adapts to the living environment within the cells of host insects. Future functional studies of *Wolbachia* will reveal whether they have an association with the virulence variation of the BPH.

We generated a list of candidate differentially expressed genes that were potentially involved in the symbiotic microorganisms of the BPH and provided a starting point for further exploring the molecular basis of symbiosis among the two BPH populations with different virulence.

## Conclusions

We present here the first comprehensive evaluation of the fat body transcriptome of the BPH using high-throughput sequencing and a comparative expression analysis between two BPH populations with different virulence levels. A total of 42,621 unique unigenes were obtained, which might provide a major genomic resource for investigating the fat body of the BPH. GO analysis of all annotated unigenes showed a similar distribution of gene functions between them. The 317 unigenes showing homology with yeast-like symbionts were predicted to belong to 12 genera of fungal species. Furthermore, 7,860 differentially expressed genes were identified in the fat bodies of the two populations. GO annotation and KEGG pathway enrichment analysis indicated that the differentially expressed genes related to metabolism and immunity were active in the fat bodies. The exploration of these differentially expressed genes suggested that these genes had important functions and might be associated with BPH virulence traits. Finally, 105 differentially expressed genes from yeast-like symbionts and *Wolbachia* were identified. Given important roles of genes related to metabolism and immunity and microbial symbionts in BPH virulence variation [11], [14], [16], [18], [19], the results provide a valuable resource for future investigations into the molecular mechanisms responsible for virulence variation in the BPH, which should afford new strategies for controlling this important agricultural pest.

## Supporting Information

Figure S1 **Length distribution of unigenes in fat body transcriptomes of brown planthopper** ***Nilaparvata lugens*** **populations.** The *x*-axis shows the calculated lengths of the unigenes in the fat body library and the *y*-axis shows the number of unigenes. (A) avirulent TN1 population. (B) virulent M population.
(TIF)

Table S1 **Primers used in QRT-PCR to validate differentially expressed genes.**
(XLSX)

Table S2 **Annotation of GenBank database searches.**
(XLS)

Table S3 **Top hits obtained by BLASTX searches in the NCBI database for species distribution.**
(XLSX)

Table S4 **Gene ontology (GO) analysis of the fat body transcriptomes of TN1 and Mudgo (M) populations.**
(XLS)

Table S5 **Possible yeast-like symbionts and** ***Wolbachia*** **genes in the fat body transcriptomes of two populations. M, Mudgo.**
(XLS)

Table S6 **Verification of differentially expressed genes between two populations by QRT-PCR.**
(XLSX)

Table S7 **Gene ontology (GO) analysis of differentially expressed genes between TN1 and Mudgo (M) populations.**
(XLS)

Table S8 **Differentially expressed genes related to metabolism between TN1 and Mudgo (M) populations.**
(XLSX)

Table S9 **Differentially expressed genes related to immunity between TN1 and Mudgo (M) populations.**
(XLS)
