# Supplementary figures and images for: Transcriptome Analysis of Fat Bodies from Two Brown Planthopper (Nilaparvata lugens) Populations with Different Virulence Levels in Rice
Source: PLoS One. 2014 Feb 12;9(2):e88528. doi: 10.1371/journal.pone.0088528 (PMC3922922; doi:10.1371/journal.pone.0088528)

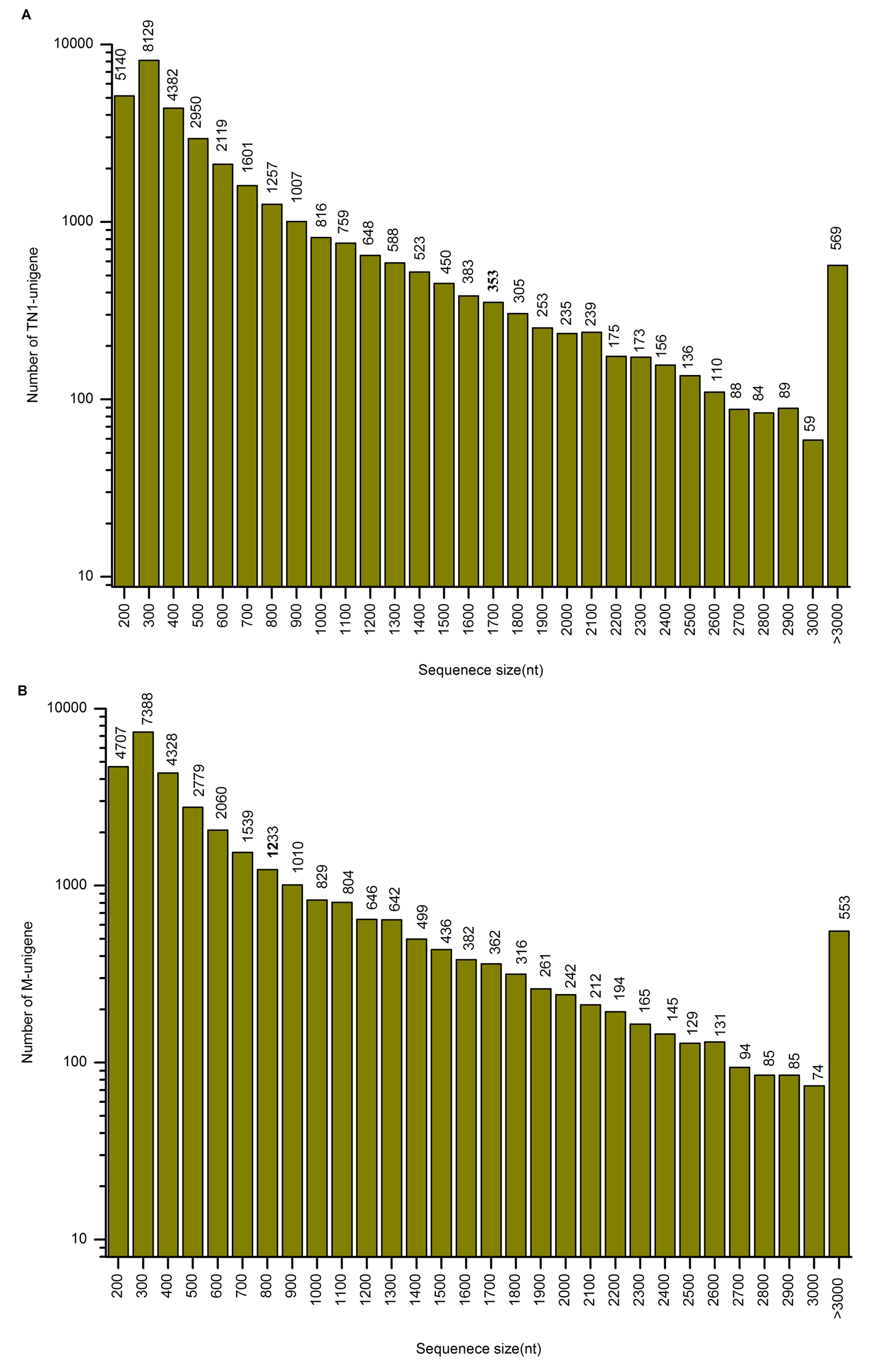

Supplement: Figure S1 — Length distribution of unigenes in fat body transcriptomes of brown planthopper Nilaparvata lugens populations. The x-axis shows the calculated lengths of the unigenes in the fat body library and the y-axis shows the number of unigenes. (A) avirulent TN1 population. (B) virulent M population. (TIF) [file pone.0088528.s001.tif]
